# Sampling bias and incorrect rooting make phylogenetic network tracing of SARS-COV-2 infections unreliable

**DOI:** 10.1073/pnas.2007295117

**Published:** 2020-05-07

**Authors:** Carla Mavian, Sergei Kosakovsky Pond, Simone Marini, Brittany Rife Magalis, Anne-Mieke Vandamme, Simon Dellicour, Samuel V. Scarpino, Charlotte Houldcroft, Julian Villabona-Arenas, Taylor K. Paisie, Nídia S. Trovão, Christina Boucher, Yun Zhang, Richard H. Scheuermann, Olivier Gascuel, Tommy Tsan-Yuk Lam, Marc A. Suchard, Ana Abecasis, Eduan Wilkinson, Tulio de Oliveira, Ana I. Bento, Heiko A. Schmidt, Darren Martin, James Hadfield, Nuno Faria, Nathan D. Grubaugh, Richard A. Neher, Guy Baele, Philippe Lemey, Tanja Stadler, Jan Albert, Keith A. Crandall, Thomas Leitner, Alexandros Stamatakis, Mattia Prosperi, Marco Salemi

**Affiliations:** ^a^Emerging Pathogens Institute, University of Florida, Gainesville, FL 32609;; ^b^Department of Pathology, Immunology and Laboratory Medicine, University of Florida, Gainesville, FL 32610;; ^c^Institute for Genomics and Evolutionary Medicine, Temple University, Philadelphia, PA 19122;; ^d^Department of Epidemiology, University of Florida, Gainesville, FL 32610;; ^e^Department of Microbiology, Immunology and Transplantation, Rega Institute for Medical Research, Clinical and Epidemiological Virology, Katholieke Universiteit Leuven, 3000 Leuven, Belgium;; ^f^Center for Global Health and Tropical Medicine, Instituto de Higiene e Medicina Tropical, Universidade Nova de Lisboa, 1349-008 Lisboa, Portugal;; ^g^Spatial Epidemiology Laboratory, Université Libre de Bruxelles, 1050 Bruxelles, Belgium;; ^h^Network Science Institute, Northeastern University, Boston, MA 02115;; ^i^Department of Medicine, University of Cambridge, Cambridge CB2 3QG, United Kingdom;; ^j^Centre for the Mathematical Modelling of Infectious Diseases, London School of Hygiene & Tropical Medicine, London WC1E 7HT, United Kingdom;; ^k^Department of Infectious Disease Epidemiology, London School of Hygiene & Tropical Medicine, London WC1E 7HT, United Kingdom;; ^l^Division of International Epidemiology and Population Studies, Fogarty International Center, National Institutes of Health, Bethesda, MD 20892;; ^m^Department of Computer & Information Science & Engineering, University of Florida, Gainesville, FL 32611;; ^n^Department of Informatics, Craig Venter Institute, La Jolla, CA 92037;; ^o^Division of Vaccine Discovery, La Jolla Institute for Immunology, La Jolla, CA 92037;; ^p^Department of Pathology, University of California San Diego, La Jolla, CA 92093;; ^q^Unité Bioinformatique Evolutive, Centre de Bioinformatique, Biostatistique et BiologieIntégrative (C3BI)–USR 3756 CNRS and Institut Pasteur, 75015 Paris, France;; ^r^State Key Laboratory of Emerging Infectious Diseases, School of Public Health, The University of Hong Kong, Hong Kong SAR, China;; ^s^Department of Biomathematics, David Geffen School of Medicine, University of California, Los Angeles, CA 90095;; ^t^Department of Biostatistics, David Geffen School of Medicine, University of California, Los Angeles, CA 90095;; ^u^Department of Human Genetics, David Geffen School of Medicine, University of California, Los Angeles, CA 90095;; ^v^KwaZulu-Natal Research Innovation and Sequencing Platform, University of KwaZulu-Natal, Durban 4041, South Africa;; ^w^Department of Epidemiology and Biostatistics, School of Public Health, Indiana University, Bloomington, IN 47405;; ^x^Max F. Perutz Laboratories, Center for Integrative Bioinformatics Vienna (CIBIV), Medical University of Vienna, University of Vienna, 1030 Vienna, Austria;; ^y^Department of Integrative Biomedical Sciences, Institute of Infectious Disease and Molecular Medicine, University of Cape Town, Cape Town 7925, South Africa;; ^z^Vaccine and Infectious Disease Division, Fred Hutchinson Cancer Research Center, Seattle, WA 98109;; ^aa^Department of Zoology, University of Oxford, Oxford OX1 3PS, United Kingdom;; ^bb^Department of Epidemiology of Microbial Diseases, Yale School of Public Health, New Haven, CT 06510;; ^cc^Biozentrum, University of Basel, 4056 Basel, Switzerland;; ^dd^Department of Biosystems Science and Engineering, Swiss Federal Institute of Technology, 4058 Basel, Switzerland;; ^ee^Department of Microbiology Tumor and Cell Biology, Karolinska Institutet, 17177 Stockholm, Sweden;; ^ff^Department of Biostatistics & Bioinformatics, Computational Biology Institute, Milken Institute School of Public Health, George Washington University, Washington, DC 20052;; ^gg^Theoretical Biology & Biophysics, Los Alamos National Laboratory, Los Alamos, NM 87545;; ^hh^Computational Molecular Evolution Group, Heidelberg Institute for Theoretical Studies, 69118 Heidelberg, Germany;; ^ii^Institute for Theoretical Informatics, Karlsruhe Institute of Technology, 76131 Karlsruhe, Germany

There is obvious interest in gaining insights into the epidemiology and evolution of the virus that has recently emerged in humans as the cause of the coronavirus disease 2019 (COVID-19) pandemic. The recent paper by Forster et al. (1) analyzed 160 severe acute respiratory syndrome coronavirus (SARS-CoV-2) full genomes available (https://www.gisaid.org/) in early March 2020. The central claim is the identification of three main SARS-CoV-2 types, named A, B, and C, circulating in different proportions among Europeans and Americans (types A and C) and East Asians (type B). According to a median-joining network analysis, variant A is proposed to be the ancestral type because it links to the sequence of a coronavirus from bats, used as an outgroup to trace the ancestral origin of the human strains. The authors further suggest that the “ancestral Wuhan B-type virus is immunologically or environmentally adapted to a large section of the East Asian population, and may need to mutate to overcome resistance outside East Asia.” There are several serious flaws with their findings and interpretation. First, and most obviously, the sequence identity between SARS-CoV-2 and the bat virus is only 96.2%, implying that these viral genomes (which are nearly 30,000 nucleotides long) differ by more than 1,000 mutations. Such a distant outgroup is unlikely to provide a reliable root for the network. Yet, strangely, the branch to the bat virus, in figure 1 of their paper, is only 16 or 17 mutations in length. Indeed, the network seems to be misrooted, because (see their SI Appendix, figure S4) a virus from Wuhan from week 0 (24 December 2019) is portrayed as a descendant of a clade of viruses collected in weeks 1 to 9 (presumably from many places outside China), which makes no evolutionary (2) or epidemiological sense (3).

As for the finding of three main SARS-CoV-2 types, we must underline that finding different lineages in different countries and regions is expected with any RNA virus experiencing founder effects (2). According to Forster et al.’s (1) own analysis, a single synonymous mutation (nucleotide change in a gene that does not result in a modified protein) distinguishes type A from type B, while one nonsynonymous mutation (resulting in a protein with a single amino acid change) separates types A and C, and another one separates types B and C. Given SARS-CoV-2’s fast evolutionary rate, random emergence of new mutations is entirely expected, even in a relatively short timeframe (4). When a viral strain is introduced and spreads in a new population, such random mutations can be propagated without them being selected or advantageous, due to founder effects. The fact that SARS-CoV-2 sequences show some geographical clustering is not new and is nicely and interactively shown on Nextstrain (5), but this cannot be used as a proof of biological differences unless backed by solid experimental data (6). This is particularly true for the work of Forster et al., since their findings are based on a nonrepresentative dataset of 160 genomes, with no significant correlation between prevalence of confirmed cases and number of sequenced strains per country (7, 8). The essential role of representative sampling is well documented in the literature (9), but was not acknowledged by the authors, who, instead, claim that their “network faithfully traces routes of infections for documented [COVID-19] cases,” without taking into consideration missing viral diversity, or evaluating multiple transmission hypotheses that would be consistent with sequence data, or even providing any support on the robustness of the branching pattern in their network. Ultimately, no firm conclusion should be drawn without evaluating the probability of alternative dissemination routes.

The inappropriate application and interpretation of phylogenetic methods to analyze limited and unevenly sampled datasets begs for restraint about origin, directionality, and early clade/lineage inference of SARS-CoV-2. We feel the urgency to reframe the current debate in more rigorous scientific terms, given the dangerous implications of misunderstanding the true dispersal dynamics of SARS-CoV-2 and the COVID-19 pandemic.
